# The epidemiology of intestinal protozoa in the Israeli population based on molecular stool test: a nationwide study

**DOI:** 10.1128/spectrum.00616-24

**Published:** 2024-07-16

**Authors:** Avi Peretz, Maya Azrad, Shifra Ken- Dror, Merav Strauss, Dana Sagas, Miriam Parizada, Shulamit Loewnthal, Doron Amichay, Nili Ben Horin, Yotam Shenhar, Orli Sagi, Elina Bazarsky, Sharon Amit, Eliezer Schwartz

**Affiliations:** 1 Clinical Microbiology Laboratory, Tzafon Medical Center, Poriya, Israel, affiliated with Azrieli Faculty of Medicine, Bar Ilan University, Israel, Safed; 2 The Azrieli Faculty of Medicine, Bar Ilan University, Safed, Tiberias, Israel; 3 Clinical Microbiology Laboratory, Central Laboratories Haifa and Western Galilee, Clalit Health Services, Nesher, Israel; 4 Clinical Microbiology Laboratory, Emek Medical Center, Clalit Health Services, Afula, Israel; 5 Maccabi Health Services, Central Laboratories, Rehovot, Israel; 6 Central Laboratories, Clalit Health Services, Tel Aviv, Israel; 7 Leumit Central Laboratories, Or Yehuda, Israel; 8 Microbiology Laboratory, Soroka University Medical Center, Beer-Sheva, Israel; 9 Faculty of health science, Ben-Gurion University, Beer-Sheva, Israel; 10 Clinical Microbiology, Sheba Medical Center, Ramat Gan, Israel; 11 Faculty of Medicine, Tel Aviv University, Tel Aviv, Israel; 12 The Institute of Tropical Medicine, Sheba Medical Center, Ramat Gan, Israel; Institut Pasteur, Paris, France

**Keywords:** intestinal protozoa, Israeli population, stool microscopy analysis, multiplex RT-PCR, *D. fragilis*, *Blastocystis spp*, *G. lamblia*, *E. histolytica*

## Abstract

Stool examination using microscopy was the traditional method for the diagnosis of intestinal parasites. Recently, the use of molecular tests to identify stool protozoa has become the main tool used in most clinical laboratories in Israel. This study aimed to evaluate the prevalence of intestinal parasites in Israel and to compare this prevalence in laboratories that use molecular tests vs a laboratory that uses microscopy. Samples collected from January to October 2021 at seven laboratories were analyzed by real-time PCR (RT-PCR) or by microscopy. The multiplex panel included the following pathogens: *Giardia lamblia*, *Entamoeba histolytica*, *Cryptosporidium spp.*, *Cyclospora*, *Dientamoeba fragilis*, and *Blastocystis spp*. Overall, 138,415 stool samples were tested by RT-PCR and 6,444 by microscopy. At least one protozoa species was identified in 28.4% of the PCR-tested samples compared to 4.6% of the microscopy-tested samples. *D. fragilis* was the most common PCR-identified species (29%). *D. fragilis*, *G. lamblia*, and *Cryptosporidium spp*. were mainly found in pediatric population, while *Blastocystis spp*. was most prevalent among adults (*P* < 0.001). In a sub-cohort of 21,480 samples, co-infection was found in 4,113 (19.15%) samples, with *Blastocystis spp*. and *D. fragilis* being the most common (14.9%) pair. Molecular stool testing proved more sensitive compared to microscopy. *D. fragilis* was the most commonly detected pathogen. The above profile was identified during the COVID pandemic when traveling was highly restricted and most likely represents the locally circulating protozoa.

**IMPORTANCE:**

This study sheds light on the prevalence of stool parasites in Israel. Additionally, this study indicates that the shift from microscope analysis to molecular tests improved protozoa diagnosis.

## INTRODUCTION

Molecular techniques have been introduced to the clinical microbiology laboratory in the last two decades (1). These tests have higher sensitivities and specificities and faster pathogen identification turn-around times compared to traditional tests (2). Furthermore, nucleic acid detection provides a solution for the identification of viruses, fastidious bacteria, and pathogens with special growth conditions, such as *Chlamydia trachomatis* and *Clostridioides difficile*. In recent years, the identification of infectious diseases has shifted from traditional to syndromic diagnosis, which was enabled by the simultaneous detection of several pathogens (viruses, fungi, parasites, and bacteria) using molecular assays (3). Nevertheless, the use of molecular tests in parasitology is still limited, and the cornerstone of parasite infection diagnosis is still microscopy-based (4). While this methodology is time-consuming and requires high-level skilled personnel, it is still the most accurate means of identification, as not all human parasites are covered by molecular tests. Additionally, microscopy is cost-effective and suitable for resource-limited countries, where the prevalence of parasite infections is high (5).

Parasitic infections pose a major health concern to the world population, resulting in the death of 200,000 individuals each year (6). Intestinal parasites primarily cause diarrhea but have also been associated with impaired cognitive development and iron deficiency anemia (7). Protozoa is the dominant parasitic group causing intestinal infections and mainly elicit persistent abdominal symptoms (8, 9) and occasionally cause acute diarrhea.

The most common protozoa involved in intestinal infection are *Giardia lamblia* (*G. lamblia*), *Entamoeba histolytica*/*dispar* (*E. histolytica*/*dispar*), and *Cryptosporidium parvum* (*C. parvum*) (10). These parasites are transmitted via the fecal-oral route, through consumption of infected food, water or by direct contact with infected individuals or animals (11). Environmental, social, and geographical factors affect the prevalence and distribution of these parasites (12).

Epidemiological information regarding protozoa circulating in humans in Israel is very limited and has been collected in microscopy-based analyses (13, 14). Yet, molecular tests have recently become the main tool used in the major Health Maintenance Organization (HMO) laboratories in Israel for the diagnosis of enteric infections, specifically intestinal protozoa.

The current study assessed the prevalence of intestinal protozoa among symptomatic patients in Israel and compared this prevalence between molecular and microscopy-based stool test results. As the study was conducted in 2021 while Israel was in lockdown due to the COVID-19 pandemic, the prevalence and distribution of parasites most likely reflect the real epidemiology of protozoal infection in Israel.

## MATERIALS AND METHODS

### Study design

Results of stool analysis tests were prospectively collected from seven laboratories representing the three largest HMOs in the State of Israel (“Clalit Health Service” − 2 laboratories, “Maccabi Health Service” − one laboratory, and “Leumit Health Service” − one laboratory) and from the laboratories of three hospitals servicing inpatients and outpatients and that also accept community samples (Emek Medical Center, Soroka University Medical Center, and Sheba Medical Center) during January and October 2021. These laboratories captured data of about ~7 million citizens (about 75% of the entire population), living in the north, center, and south of Israel.

Stool tests were obtained from symptomatic patients; each unique patient ID number was counted only once. Clinical information and the reason for testing were not available for this study.

Out of the seven laboratories, six performed molecular testing, and one laboratory (of “Leumit” HMO) performed microscopic analysis only.

The study was approved by a Helsinki committee Institutional Review Board (IRB), approval number 0884–23-SMC-D. The need for informed consent was waived. All procedures were performed in compliance with relevant laws and institutional guidelines and have been approved

### Molecular testing

DNA was extracted from stool samples using the STARMag Universal Cartridge kit (Seegene, Duesseldorf, Germany) with the Hamilton Microlab Starlet platform (Hamilton Company, Nevada, USA). After DNA extraction, parasites were detected using the Allplex GI parasite assay (Seegene, Seoul, South Korea), according to the manufacturer’s instructions. The kit is a one-step real-time PCR that simultaneously identifies six parasites: *Blastocystis spp*. (*Blastocystis spp*.), *Cryptosporidium spp*., *Cyclospora cayetanensis* (*C. cayetanensis*), *Dientamoeba fragilis*, *E. histolytica*, and *G. lamblia*. None of the laboratories were programed to detect *E. dispar*. Results were interpreted using the automatic Seegene results processing software.

### Microscopic analysis

Stool samples were processed with Mini Parasep kit (Apacor Ltd., Wokingham, UK) containing formalin, triton X, and ethyl acetate, according to the manufacturer’s instructions. The fecal sediments were examined under a light microscope by a parasitologist. In the case of *Cryptosporidium spp*. detection, an acid-fast staining was performed.

### Statistics

*χ*^2^ test was used to analyze differences in categorical variables (age and gender). Independent samples *t* test was used to analyze differences in the mean and median age of adults and children. A *P* value < 0.05 was considered statistically significant. The data were analyzed using the RStudio version 2021.09.0 Build 351.

## RESULTS

During the first 10 months of 2021, 144,859 stool samples were collected from patients, 59% of whom were female and 41% male. The laboratories of “Clalit Health Service” collected 66,342 samples, the laboratory of “Maccabi Health Service” collected 40,063 samples, and “Leumit Health Service” laboratory collected 6,444 samples. The Emek Medical Center’s laboratory collected 13,300 samples, Soroka University Medical Center’s laboratory collected 12,553 samples, and Sheba Medical Center’s laboratory collected 6,157 samples. Molecular testing was performed on 138,415 samples, while 6,444 samples were analyzed by microscopy.

Parasites were detected in 27.3% of the samples (39,606/144,859). Of the 138,415 samples tested by PCR, 28.4% (39,310) were positive for at least one protozoa species, whereas microscopy found at least one species in 4.6% (296) of the samples (Table 1).

**TABLE 1:** Distribution of protozoa in stool samples tested by PCR compared to samples tested by microscopy^*b*^

Protozoa	PCR *N* = 138,415		Microscopy *N* = 6,444	
	*n* (% of total samples)	% of positive samples	*n* (% of total samples)	% of positive samples
*D. fragilis* (*N* = 71,414*)^a^*	20,710 (29^*a*^)	52.7	0 (0)	0
*Blastocystis spp.* (*N* = 71,414)^*a*^	12,426 (17.4^*a*^)	31.6	115 (1.8)	38.8
*G. lamblia*	4,983 (3.6)	12.6	77 (1.2)	26.0
*Cryptosporidium spp.*	969 (0.7)	2.5	6 (0.09)	2.0
*E. histolytica*	16 (0.01)	0.04	0 (0)	0
*C. cayetanensis*	3 (0.002)	0.007	0 (0)	0
*E. histolytica/dispar*	ND		6 (0.09)	2.0
*Endolimax nana*	ND		19 (0.3)	6.4
*Chilomastix mesnili*	ND		1 (0.015)	0.3
*Entamoeba coli*	ND		58 (0.9)	19.6
*Iodamoeba butschlii*	ND		6 (0.09)	2.0
Total	39,310 (28.4)		296 (4.6)	

^*a*^ *D. fragilis* and *Blastocystis spp.* were not reported by all laboratories, and thus, for calculation of these pathogens’ prevalence, the total samples’ number is 71,414.

^*b*^ ND = not done.

The most common protozoa species found by PCR was *D. fragilis* (29%), followed by *Blastocystis spp.* (17.4%), *G. lamblia* (3.6%), and *Cryptosporidium spp.* (0.7%). Interestingly, *E. histolytica* was detected in 16 cases only (0.01%), and *Cyclospora* was diagnosed in three patients (0.002%). There were no differences in parasite distribution between laboratories from different geographic areas (data not shown).

In microscopy-tested samples, *Blastocystis spp.* (1.8%) and *G. lamblia* (1.2%) were the most prevalent species, while *D. fragilis* was not detected at all. Additionally, several other protozoa not included within the multiplex PCR, such as *Entamoeba coli* and *Endolimax* nana, were found by microscopy, albeit in very small numbers (Table 1).

Further analysis was performed on a cohort of 21,480 patients from “Clalit” since it is the largest HMO in Israel and covers the widest geographic areas. Out of these 21,480 samples, two or more concurrent protozoa species were detected in 4,113 (19.15%) samples (Table 2).

**TABLE 2:** Number of protozoa species in stool samples (*N* = 21,480)

Number of parasites per sample	*n* (%)
1	17,367 (80.85)
2	3873 (18.05)
3	237 (1.1)
4	3 (0.01)

**Fig 1:**
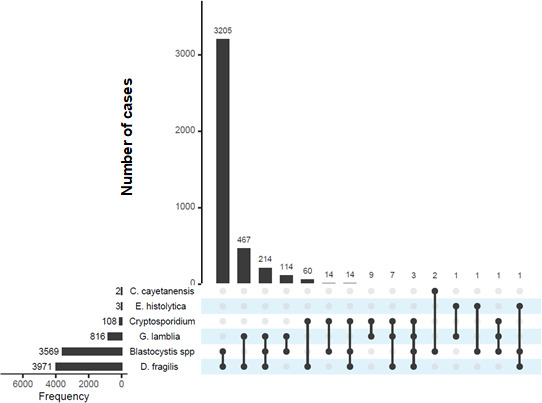
Parasite co-infections. The graph presents the number of samples with parasite co-infections, with the species pairs indicated below the graph (*N* = 4,113).

In these cases of multiple infections, the most common co-infections were Blastocystis spp. with *D. fragilis* (14.9%) and *G. lamblia* with *D. fragilis* (2.2%; Fig. 1).

The mean age of adults (>18 years) and pediatric patients (≤18 years) in this cohort was 48.8 years and 6.1 years, respectively (Table 3). Most adults (61.1%) were female, while infection was more evenly distributed between male and female children.

**TABLE 3:** Demographic characteristics and parasite distribution in the adult and pediatric population

	Pediatric (*N* = 11,619)	Adults (*N* = 9,861)	Total (*N* = 21,480)	*P* value
Female (*n*, %)	5,641 (48.5)	6,025 (61.1)	11,666 (54.3)	<0.001
Age (years) – mean ± SD	6.1 ± 4.1	48.8 ± 18.6	25.7 ± 24.9	<0.001
Median (years)	5.1	44.8	13.3	
Parasite, *n* (%)				<0.001
*C. cayetanensis*	0 (0)	3 (0.03)	3 (0.01)	0.097
*D. fragilis*	8,136 (70.0)	4,604 (46.7)	12,740 (59.3)	<0.001
*Blastocystis spp.*	2,131 (18.3)	4,802 (48.7)	6,933 (32.3)	<0.001
*G. lamblia*	1,046 (9.0)	386 (3.9)	1,432 (6.7)	<0.001
*Cryptosporidium spp.*	306 (2.6)	59 (0.6)	365 (1.7)	<0.001
*E. histolytica*	0 (0)	7 (0.1)	7 (0.03)	0.004

**Fig 2:**
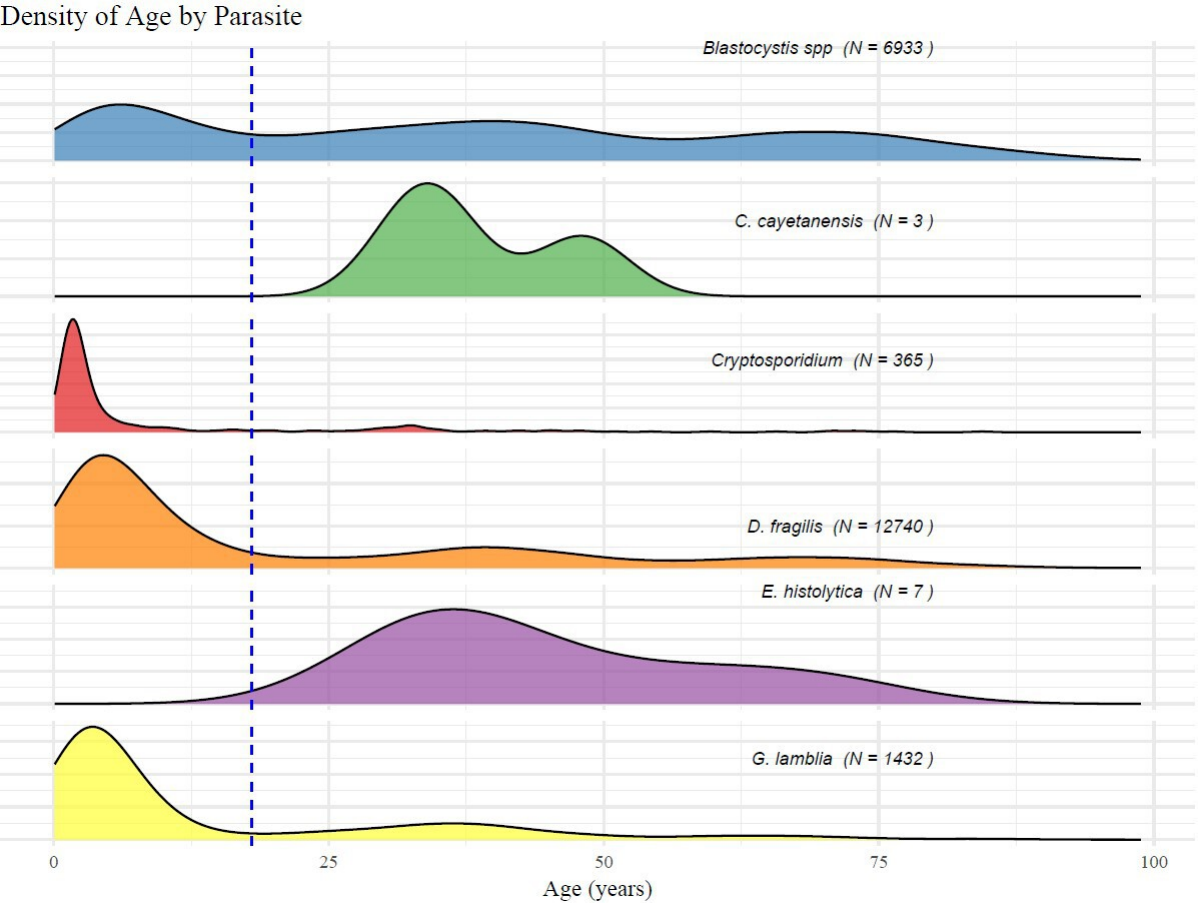
Prevalence of protozoa infection by age (*N* = 21,480). The graph presents the number of samples carrying each indicated parasite species in relation to patients’ age.

The distribution of the different protozoa seemed to be an age-dependent. As can be seen in Table 3 and Fig. 2, *D. fragilis*, *G. lamblia*, and *Cryptosporidium spp.* were mainly found in the pediatric population (*P* < 0.001 for each parasite), while *Blastocystis spp.* was more prevalent among adults (*P* < 0.001). *C. cayetanensis* and *E. histolytica* were only detected in a small number of adults and probably can be all imported.

## DISCUSSION

This was the first nationwide study of the epidemiology of intestinal parasites in Israel that covered almost the entire local population and was based on molecular diagnosis compared to microscopy. The study was conducted during the COVID pandemic when traveling abroad was highly restricted; thus, the findings most likely primarily represent the protozoa circulating inside Israel without imported parasites.

The analysis found an increased number of positive samples in labs using molecular compared to microscopy testing (28.4% vs 4.6%, respectively). This finding was not surprising, as other studies have demonstrated increased identification of GI pathogens, in general, and of parasites, specifically, in stool samples analyzed by molecular tests compared to microscopy (15, 16). Thus, there is no doubt that the sensitivity of molecular assays is much higher than the traditional methods for parasite testing in stool samples.

The two most common protozoa detected in the current study were *D. fragilis* and *Blastocystis* spp. This finding correlates with the results of a previous 2022 report from Israel, in which *D. fragilis* and *Blastocystis spp.* were the most detected parasites in molecular stool testing of travelers and non-travelers (8). Yet, there is an ongoing debate regarding the role of these species as pathogens (17, 18).

While the high rate of *Blastocystis* spp. is common, even when using microscopy-based diagnosis, the high rate of *D. fragilis* in the current study was a bit surprising. *D. fragilis* was the most dominant finding, accounting for 29% of samples tested using molecular methods as compared to 0% at the one center that used microscopy for diagnosis (Table 1). Similar rates were reported in other countries, such as Finland (19) and Italy (20). This may have been due to the enhanced sensitivity of PCR for *D. fragilis*, among other parasites (21, 22). *D. fragilis* is a very fragile protozoa (hence its name), and it cannot be detected by microscopy as it is already destroyed by arrival to the microscope. In contrast, its DNA can still be detected by molecular tests.

Since patients in the present cohort were most likely symptomatic, the high *D. fragilis* rate calls to revisit its role as a pathogen. In fact, a recent study demonstrated a correlation between the eradication of *D. fragilis* from the stool and the achievement of a clinical cure (19).

*G. lamblia*, once considered the most common parasitic pathogen in the United States, was found in only 3.6% of cases. Similarly, a study in New York covering 9,402 samples found *G. lamblia* in 3.3% of the samples (16). A 2022 analysis in Israel found a 1.5% prevalence for this species; however, the study was performed in one geographic area in Israel (8).

*Cryptosporidium spp.*, a pathogen that was difficult to diagnose before the PCR era (22), were identified in close to 1,000 patients (0.7%) in the current cohort*. Cryptosporidium* genotypes and *Cryptosporidium partum* subtypes were shown to have different epidemiology and clinical spectra (23). Since the present work did not include data regarding clinical presentation and epidemiology, a future study should be performed to collect such data, including protozoa genotypes.

Other important aspects of this study were the negative findings, such as the almost complete disappearance of *E. histolytica* (0.01%) which used to be endemic in Israel (14). As *E. histolytica* cannot be differentiated by microscopy from the nonpathogenic *E. dispar*, thus, its disappearance can be attributed to a more accurate diagnosis and elimination of false *E. histolytica* cases (which were actually *E. dispar*). The low rate of *E. histolytica* may reflect a true change in ameobiasis epidemiology in Israel due to the improved economic situation over time, as well as improved hygiene. Testing for *E. dispar* could point us whether this protozoan truly exists in Israel and thus could explain the positive cases of *E. histolytica* before the PCR era. However, *E. dispar* detection is not available in our molecular stool analysis platform.

*Cyclospora* is a relatively newly discovered pathogen that has never been reported in Israel before this study. Thus, the three detected cases which were found among adults only may have been imported.

An additional feature attributed to molecular assays for stool analysis is the increased sensitivity for co-infections. In the current study, co-infections were detected in 19.1% of a 21,480-sample subset. A similar rate (24%) was reported in a study from Madrid (22). According to a recent review, more than one pathogen was found in 20%−50% of positive stool samples tested by molecular assays (24). The most frequent co-infection in the current study was of *Blastocystis* spp. and *D. fragilis* (14.9%). A previous study in Israel reported a 23.8% co-infection rate among 1,359 samples, with *Blastocystis spp.* and *D. fragilis* being the most common (21.9%) combination (8). This combination was also the most prevalent co-infection (24%) found in a study performed on a cohort of 756 patients in Italy (25) and was identified in 33.6% of a 13,983-stool sample set tested by Burgana et al. (26). Similarly, a study from Turkey reported this co-infection rate to be 23.7% (27)

A major limitation of multiplex molecular testing is its identification of protozoa that are included in its predefined panel. Indeed, the single lab in this study that used microscopy identified several protozoa such as *Entamoeba coli* and *Endolimax nana* that were not included in the panel. In addition, helminths are not included at all in this multiplex test.

Another limitation that clinicians should keep in mind is the fact that different assays of multiplex molecular stool testing constitute a versatile list of detected pathogens; thus, negative results should be interpreted according to the manufacturer menu.

Although detailed demographic data were not collected in this study, the ages of a large number of patients were available, which enabled evaluation of the distribution of different protozoa by age group. *D. fragilis*, *G. lamblia*, and *Cryptosporidium spp.* were the dominant parasites in the pediatric population, while *Blastocystis spp.* was more common among adults. *D. fragilis* was shown to be commonly detected in the stools of children in Denmark in 2013 (28). Another study from the Netherlands also found a high (70%) prevalence of *D. fragilis* among pediatric patients (29). In a similar study conducted in Turkey, *D. fragilis* was detected more frequently in children, particularly among individuals between the ages of 10 and 19 (27)

This work demonstrated that molecular test is much more sensitive compared to microscopic analysis. Yet, while stool PCR testing has dramatically increased the sensitivity of protozoa detection, the actual test sensitivity remains unknown. More specifically, negative results do not exclude parasitic infection, especially since a limited panel of protozoa is included in each test. Protozoa are dominant pathogens mainly in cases of persistent abdominal symptoms even without diarrhea (9). Many cases diagnosed as irritable bowel syndrome due to negative stool results in fact could be cured with anti-parasitic treatment (30).

This study had several limitations. First, clinical patient data and data regarding treatment, which could have impacted results, were not collected. Second, the same samples were not tested in parallel by both PCR and microscopy, which would have given a more accurate picture concerning the reliability of molecular testing as compared to the gold standard. Nevertheless, the large sample size for each method support the validity of the study results.

In conclusion, the use of multiplex PCR stool testing increased the sensitivity of protozoa detection in symptomatic patients. Israel, despite being a high-income country, demonstrated the possible role of protozoa infection in patients with GI complaints. *D. fragilis* seems to be an emerging pathogen, and further studies are needed to elucidate its role as an enteric pathogen.
